# NOX4-Derived ROS Mediates TGF-*β*1-Induced Metabolic Reprogramming during Epithelial-Mesenchymal Transition through the PI3K/AKT/HIF-1*α* Pathway in Glioblastoma

**DOI:** 10.1155/2021/5549047

**Published:** 2021-06-27

**Authors:** Xiangsheng Su, Yihang Yang, Changfa Guo, Rui Zhang, Shicheng Sun, Yanjun Wang, Qiujiang Qiao, Yibing Fu, Qi Pang

**Affiliations:** ^1^Department of Neurosurgery, Shandong Provincial Hospital, Cheeloo College of Medicine, Shandong University, Jinan, Shandong 250012, China; ^2^Department of Obstetrics and Gynecology, Shandong Provincial Hospital, Cheeloo College of Medicine, Shandong University, Jinan, Shandong 250012, China

## Abstract

Current studies on tumor progression focus on the roles of cytokines in the tumor microenvironment (TME), and recent research shows that transforming growth factor-*β*1 (TGF-*β*1) released from TME plays a pivotal role in tumor development and malignant transformation. The alteration in cellular metabolism is a hallmark of cancer, which not only provides cancer cells with ATP for fuel cellular reactions, but also generates metabolic intermediates for the synthesis of essential cellular ingredients, to support cell proliferation, migration, and invasion. Interestingly, we found a distinct metabolic change during TGF-*β*1-induced epithelial-mesenchymal transition (EMT) in glioblastoma cells. Indeed, TGF-*β*1 participates in metabolic reprogramming, and the molecular basis is still not well understood. NADPH oxidases 4 (NOX4), a member of the Nox family, also plays a key role in the biological effects of glioblastoma. However, the relationship between NOX4, TGF-*β*1, and cellular metabolic changes during EMT in glioblastoma remains obscure. Here, our findings demonstrated that TGF-*β*1 upregulated NOX4 expression accompanied by reactive oxygen species (ROS) through Smad-dependent signaling and then induced hypoxia-inducible factor 1*α* (HIF-1*α*) overexpression and nuclear accumulation resulting in metabolic reprogramming and promoting EMT. Besides, inhibition of glycolysis reversed EMT suggesting a causal relationship between TGF-*β*1-induced metabolic changes and tumorigenesis. Moreover, TGF-*β*1-induced metabolic reprogramming and EMT which modulated by NOX4/ROS were blocked when the phosphoinositide3-kinase (PI3K)/AKT/HIF-1*α* signaling pathways were inhibited. In conclusion, these suggest that NOX4/ROS induction by TGF-*β*1 can be one of the main mechanisms mediating the metabolic reprogramming during EMT of glioblastoma cells and provide promising strategies for cancer therapy.

## 1. Introduction

Glioblastoma multiforme (GBM) is the most prevalent primary malignant tumors affecting the human brain with a median survival of 15 months [1]. Despite improvements in therapeutic technologies including surgical resection, adjuvant radiotherapy, and chemotherapy, patients with GBM have a poor clinical result and the molecular mechanisms responsible for the development of GBM have not yet been fully elucidated [2]. For these reasons, the identification of novel treatments to improve the available therapeutic options is essential for managing this disease.

Increasing evidence has indicated that the tumor microenvironment (TME) is key in tumor development, including glioblastoma [3, 4]. The TME comprises tumor cells, tumor stroma, blood vessels, infiltrating inflammatory cells, and various associated tissue cells. Transform growth factor-*β* (TGF-*β*), a family of cytokines including TGF-*β*1, TGF-*β*2, and TGF-*β*3, is produced by a variety of cells in a pleiotropic manner within the TME, and it is responsible for the regulation of the activity of cells within this milieu [5]. As current studies show, TGF-*β* is a main inducer of epithelial-mesenchymal transition (EMT), autophagy, immune suppression, and metastasis during cancer progression in some cancer cells [6–9]. NADPH oxidases (NOXs) are a family of heme-containing proteins, the main function of which is the production of reactive oxygen species (ROS) [10]. A total of seven NOX proteins have been identified, including NOX1-5 and DUOX1-2 [10]. These enzymes are widely expressed in numerous tissues and have various functions, including roles in cell signaling, regulation of gene expression, cell death, differentiation, and growth. Recent evidence suggests that NOX4 expression enhances in various cells include but not limited to pancreatic cancer [11], breast cancer [12], and glioblastoma cells [13]. The role of NOX4 in glioblastoma appears to be involved in growth, survival, hypoxia, angiogenesis, and radiation resistance [13–15]. And the expression and activity of NOX4 is regulated by TGF-*β*1 in many cell types [11, 12], but it is not reported that NOX4 expression is regulated by TGF-*β*1 in glioblastoma.

Epithelial-mesenchymal transition (EMT) is characterized by morphological changes which increased cell plasticity and mobility as they transition into a mesenchymal-like cell phenotype accompanied by the decreased expression of E-cadherin, claudin, occludin, and cytokeratin and the acquisition of a motile and migratory phenotype with the increased expression of N-cadherin, twist, Vimentin, fibronectin, and snail [16]. EMT is also a process of key importance during embryogenesis, organ development, and wound healing [17]. According to the present studies, TGF-*β*1 plays a critical role in EMT [18]. Metabolic reprogramming is characterized by reduced mitochondrial oxygen consumption with a shift in subcellular energy ATP production via aerobic glycolysis in the cytosol in place of the mitochondria through oxidative phosphorylation. This is the core content of the Warburg effect or aerobic glycolysis [19]. EMT is related to complex metabolic reprogramming, orchestrated by EMT transcription factors, supporting increased energy required for motility and growth under harsh environmental conditions. Recent evidence shows that the link between EMT and metabolism is mutual, and changes in metabolism can drive EMT in some cases [20]. However, how the metabolic change is directly regulated and the molecular basis between metabolic change and EMT are still largely unknown. Recently, more and more evidence indicates that elevated ROS generation in cancer cells and excessive accumulation can function as secondary signaling molecules involving tumorigenesis, angiogenesis, invasion, chemoresistance, and metabolism [21]. As a vital source of generation for ROS, NOX4 also plays an important role in the regulation of cancer metabolism. In renal cell carcinoma, NOX4 acts as a mitochondrial energetic sensor coupling cancer metabolic reprogramming with drug resistance [22]. In non-small-cell lung cancer cells, NOX4 and NOX4-generated ROS promote glycolysis and modify glutamine metabolism [23]. However, the exact functions and mechanism of NOX4 of alteration in cellular metabolism especially during EMT of glioblastoma cells remain still unclear.

To date, no link has been reported between metabolic reprogramming during EMT mediated by increased NOX4 expression in accompany with ROS treated with TGF-*β*1 in glioblastoma. In the present study, we identified NOX4 as a critical role in TGF-*β*1-induced metabolic reprogramming and EMT. We hypothesized that TGF-*β*1 drives metabolic reprogramming and aggressive cancer by enhancing NOX4 activity.

## 2. Materials and Methods

### 2.1. Cell Culture

The U87 and A172 glioblastoma cell lines were obtained from the Cell Bank of Type Culture Collection of the Chinese Academy of Sciences. Glioblastoma cells were maintained as monolayer cultures in Dulbecco's Modified Eagle's Medium (DMEM, Gibco, USA) supplemented with 10% fetal calf serum and 1% penicillin and 1% streptomycin at 37°C with 5% CO_2_.

### 2.2. Chemical Reagents

The following chemicals were used: recombinant human TGF-*β*1 (100-16A, PeproTech, USA), GKT137831 (S7171, Selleck, USA), SIS3 (S7959, Selleck, USA), PX-478 (S7612, Selleck, USA), MK-2206 (S1078, Selleck, USA), CellROX Deep Red Reagent (C10422, Invitrogen, USA), 2-NBDG (HY-116215, MedChem Express, USA), rotenone (HY-B1756, MedChem Express, USA), Dichloroacetate (HY-Y0445A, MedChem Express, USA), GSK2837808A (HY-100681, MedChem Express, USA), MitoTracker probe (BB-44113, BestBio, China), Glycolysis stress test (103020-100, Agilent, USA), and MitoStress Test (103015-100, Agilent, USA).

### 2.3. TCGA Patient Analysis

The expression data and survival data of TGF-*β*1 and NOX4 were extracted from The Cancer Genome Atlas (TCGA) database (https://cancergenome.nih.gov/) and used for further analysis, including 5 normal human brain tissue samples, 529 LGG cancer tissue samples, and 169 GBM cancer tissue samples. The process of data collection complies with all laws and regulations.

### 2.4. Tissue Specimens and Immunohistochemical (IHC)

Analysis of all liquid nitrogen frozen or formalin-fixed paraffin-embedded glioma tissue specimens (WHO I glioma, *n* = 3; WHO II glioma, *n* = 5; WHO III glioma, *n* = 5; GBM, *n* = 15) and normal brain tissues (*n* = 5) were obtained from the Department of Neurosurgery at Shandong Provincial Hospital (Jinan, Shandong Province, China) between 2013 and 2019. All samples obtained were approved by the local ethics committee. Specimens were performed using antibodies against NOX4 (ab13303, 1 : 200, Abcam, USA) and TGF-*β*1 (ab27969, 1 : 100, Abcam, USA). Biotinylated secondary antibodies (PV-9000, ZSGB-Bio, China) were used to detect the primary antibodies.

### 2.5. Immunofluorescence (IF) Analysis

Specimens (including 5 normal human brain tissue samples and 5 GBM tissue samples) and cells were performed using antibodies against NOX4 (ab13303, 1 : 200, Abcam, USA), TGF-*β*1 (ab27969, 1 : 100, Abcam, USA), Vimentin (ab92547, 1 : 200, Abcam, USA), and HIF-1*α* (20960-1-AP, 1 : 50, Proteintech, China). Fluorescent secondary antibodies (anti-rabbit IgG, SA00013-2, 1 : 500 and anti-mouse IgG, SA00013-3, 1 : 500, Proteintech, China) were used to detect the primary antibodies.

### 2.6. Western Blot Analysis

Total cell lysates for protein analysis were prepared by scraping cells in 1x Cell Lysis Buffer (Solarbio, China) supplemented with protease/phosphatase inhibitors (Solarbio, China). Protein concentration was determined according to BCA assay (Beyotime, China), and equal amounts of protein were loaded onto sodium dodecyl sulfate-polyacrylamide gel electrophoresis gel. After wet transfer to nitrocellulose membrane using the BioRad Transfer system using 1x transfer buffer and blocking with 5% nonfat dry milk in TBST for 1 h at room temperature, membranes were probed with the specific primary antibodies against NOX4 (ab13303, 1 : 2000, Abcam, USA), LDHA (ab101562, 1 : 1000, Abcam, USA), Vimentin (ab92547, 1 : 2000, Abcam, USA), GLUT1 (ab115730, 1 : 5000, Abcam, USA), PDK1 (ab202468, 1 : 2000, Abcam, USA), PI3K (32089, 1 : 1000, Abcam, USA), N-cadherin (13116, 1 : 1000, CST, USA), Smad2/3 (8685, 1 : 1000, CST, USA), p-Smad2 (18338, 1 : 1000, CST, USA), p-Smad3 (9520, 1 : 1000, CST, USA), HK2 (2867, 1 : 1000, CST, USA), AKT (4691, 1 : 1000, CST, USA), p-AKT (4060, 1 : 2000, CST, USA), E-cadherin (AF0131, 1 : 2000, Affinity Biosciences, USA), HIF-1*α* (20960-1-AP, 1 : 1000, Proteintech, China), *β*-actin (66009-1-Ig, 1 : 5000, Proteintech, China), p-PDH-E1*α* (LS-C141922-50, 1 : 1000, Lifespan, USA), PDH-E1*α* (66119-1-Ig, 1 : 5000, Proteintech, China), Tubulin (66031-1-Ig, 1 : 10000, Proteintech, China), and GAPDH (10494-1-AP, 1 : 10000, Proteintech, China) overnight at 4°C. Membranes were washed three times with TBST and probed with secondary antibodies (anti-rabbit IgG, ZB-2301, and anti-mouse IgG, ZB-2305, ZSGB-Bio, China) for 1 h at room temperature. Membranes were subsequently washed three times with TBST and scanned using Amersham Imager 680 (GE Healthcare, USA).

### 2.7. qPCR Analysis

RNA was isolated using Trizol (Invitrogen, USA) and reverse transcribed using HiScript II Q Select RT SuperMix (Vazyme, China) according to the manufacturer's instructions. Quantitative mRNA expression was determined following qPCR using SYBR Green Master Mix (Vazyme, China). The primer sequences were shown in Table 1.

### 2.8. ROS Detection

Cells were plated in 6-well plates (3 × 10^5^ cells/well). After 24 hours of preincubation, the cells were washed with PBS and then incubated at 37°C with 5% CO_2_ for 2 hours in serum-free DMEM supplemented with 5 *μ*M CellROX™ Deep Red Reagent for 10 minutes. Then, the cells were washed three times with warm PBS, followed by fluorescence measurement using ImageXpress Micro Confocal (Molecular Devices, USA).

### 2.9. Lentivirus Transfection

Stable knockdowns of NOX4 and the control genes in cells were achieved by transfection with lentivirus synthesized by Genomeditech (Shanghai, China). Cells were infected with shNOX4 or negative control lentivirus vectors, and the sequence for the shNOX4 was as follows: GGTATATCCGGAGCAATAAGC, and the negative control was as follows: TTCTCCGAACGTGTCACGT.

### 2.10. RNA Interference

Small interfering RNA (siRNA) duplexes were introduced into cells using a Lipofectamine™ 2000 (Invitrogen, USA) according to the procedure recommended by the manufacturer. Cells were harvested after 48 hours for subsequent experiments. All siRNA products are obtained from GenePharma (Shanghai, China). The siRNA sequences were shown in Table 2.

### 2.11. Measurement of Cellular Metabolism

Cells were collected and plated in Agilent Seahorse XF96 plates at a density of 5 × 10^4^ cells per well and allowed to adhere for 4 hours in a standard incubator. Cells were next equilibrated with XF Base media at 37°C for one hour in an incubator lacking CO_2_, and Seahorse assay was performed. For the glycolysis stress test, cells were serum-starved for 1 hour in glucose-free media containing treatments, and measurement of ECAR was performed before and after the sequential addition of glucose, oligomycin, and 2-DG with measurements performed. For the MitoStress Test, cells were incubated in glucose-containing media for 1 hour containing treatments, and measurements were performed before and after sequential addition of oligomycin, FCCP, and rotenone/antimycin A. Wave software and GraphPad Prism were used to analyze the dates.

### 2.12. 2-NBDG Uptake Assay

Cells were plated in 96-well plates (2 × 10^4^ cells/well). After 24 hours of preincubation with TGF-*β*1 and GKT137831, the cells were washed with PBS and then incubated at 37°C with 5% CO_2_ for a period of time in glucose-free DMEM supplemented with 50 *μ*M 2-NBDG for 30 minutes. After the incubation period, cells were washed three times with warm PBS. Images were acquired using ImageXpress Micro Confocal, and mean fluorescence intensity (MFI) of 2-NBDG was also analyzed by flow cytometry.

### 2.13. Measurement of Lactate

Lactate levels in the culture medium were measured using a Lactate Colorimetric Assay Kit (Nanjing Jiancheng Corporation, China). Lactate was released in 6-well plates for 24 hours, and then, the cell supernatant was harvested and mixed with the assay solution, and the absorbance was measured at 530 nm according to the manufacturer's instructions.

### 2.14. MitoTracker Staining

Cells were fluorescently labeled with MitoTracker probe for 1 hour under the growth condition, following three washes with PBS, and then, the images were captured by ImageXpress Micro Confocal.

### 2.15. Measurement of Pyruvate Dehydrogenase (PDH) Activity

PDH activity was examined in cell lysates using a PDH activity detection kit (Solarbio, China) with the manufacturer's recommended protocols.

### 2.16. Cell Counting Kit-8 (CCK-8) Assays

Cells were seeded in 96-well plates (1 × 10^4^ cells/well), and each sample was seeded in three replicates. After 0 hour, 24 hours, 48 hours, and 72 hours of growth, the cells were added with 10 *μ*l CCK-8 reagents (Glpbio, USA) and cultured at 37°C with 5% CO_2_ for 1 hour. The optical density (OD) absorbance of each well was measured at 450 nm using the Thermo Scientific Microplate Reader to determine the cell viability.

### 2.17. EdU Proliferation Assays

The proliferation assay was performed by EdU (5-ethynyl-2′-deoxyuridine) to measure cell proliferation. In brief, cells treated were seeded in 96-well plates (2 × 10^4^ cells/well) and cultured for 24 hours treated with or without TGF-*β*1. Subsequently, the cells were incubated in serum-free DMEM containing 50 *μ*M EdU (RiboBio, China) for 2 hours after being washed three times with PBS. Then, the cells were fixed with 4% polyformaldehyde in PBS for 30 min at room temperature. Finally, cells were incubated with Apollo staining solution and Hoechst 33342 for 30 min. The proliferation index was described as the percentage of EdU-positive cells relative to the total cell numbers. Images were obtained using ImageXpress Micro Confocal, and cells selected from three random fields were counted.

### 2.18. Migration and Invasion Assay

The migration and invasion capability of cells were evaluated using Transwell cell culture inserts (Corning Incorporated, USA). Filters coated with 10% Matrigel were used for the invasion assay. In brief, cells (1 × 10^5^) in serum-free medium were loaded into the upper chamber whereas the lower chamber was filled with standard medium. Following incubation at 37°C for 24 hours, cells on the upper surface of the membrane were gently removed. The cells that had invaded the lower surface of the membrane were fixed with 4% paraformaldehyde and stained with 0.5% crystal violet, and staining cells were photographed counted in three randomly selected fields.

### 2.19. Xenografted Tumor Model

U87 cells (approximately 1 × 10^5^) were intracranially injected into 4-week-old male athymic Nu/Nu mice obtained from Vital River Laboratories. A total of five animals per condition were used, and the animal groups were as follows: PBS group (control group), TGF-*β*1 group, and TGF-*β*1 plus GKT137831 group. Recombinant human TGF-*β*1 (10 *μ*g/ml) was intranasally administered, and GKT137831 (60 mg/kg/day) was orally administered after the glioblastoma cells were injected. Briefly, a volume of 10 *μ*l solution was delivered in nose drops (contained 0.1 *μ*g TGF-*β*1) over a period of 10 minutes, alternating between each naris every 2 minutes once a day and the same volume of PBS as control. GKT137831 was used by oral application. When the mice presented with neurological signs, they were monitored and killed. Tumor sizes were monitored by MRI per week. Tissues harvested from tumors were fixed in 10% formaldehyde overnight and then embedded in paraffin and prepared for HE stain or stored in liquid nitrogen for western blot. The expressions of related proteins were detected by western blot. All experiments using mice were approved by the ethics committee of Provincial Hospital affiliated with Shandong University.

### 2.20. Statistics Analysis

The statistical analyses were performed using the SPSS statistical software package and the GraphPad Prism statistical program. Student's *t*-test, Spearman rank correlation test, and log-rank test were used for analyzing the statistical significance of all data. The data are presented as means ± standard deviations (SD) of at least three independent experiments. Two-tailed *P* values <0.05 were considered statistically significant. ns means not significant; ^∗^*P* < 0.05; ^∗∗^*P* < 0.01; ^∗∗∗^, *P* < 0.001.

## 3. Results

### 3.1. TGF-*β*1 and NOX4 Are Highly Expressed in Glioblastoma and Associated with the Grade of Glioma

**Figure 1:**
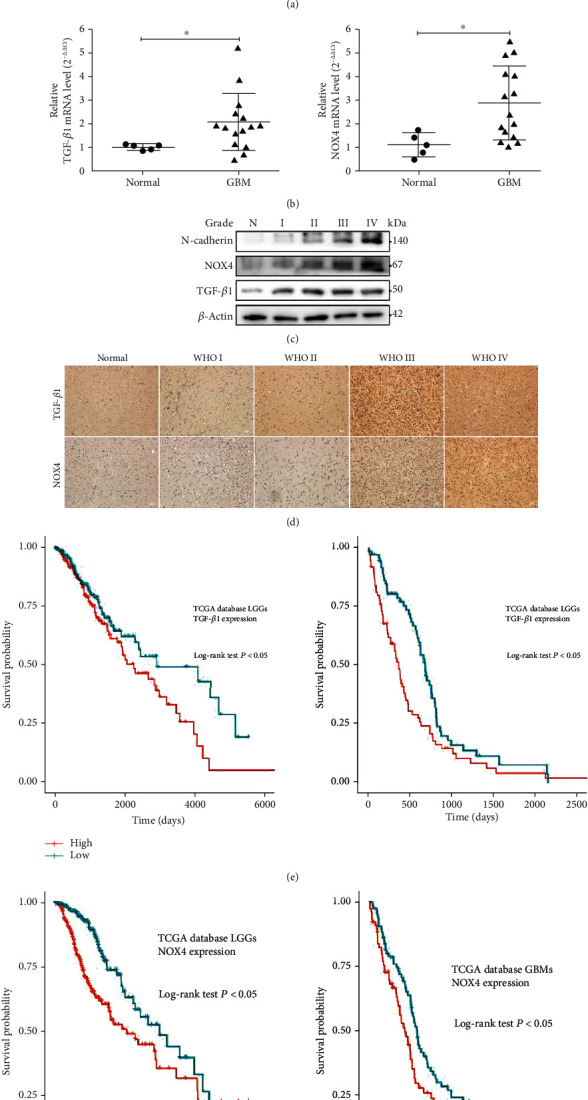
TGF-*β*1 and NOX4 are highly expressed in glioblastoma and associated with the grade of glioma. (a) Analysis of TGF-*β*1 and NOX4 expression in normal brain and glioma tissues based on TCGA database. (b) qPCR analysis of the TGF-*β*1 and NOX4 mRNA levels in the normal human brain samples and GBM tissues. (c) Western blot analysis of TGF-*β*1 and NOX4 protein expression in the normal human brain samples and glioma tissues. (d) Immunohistochemical staining for TGF-*β*1 and NOX4 protein expression in the normal human brain samples and glioma tissues. Scale bar = 50 *μ*m. (e, f) The prognostic value of TGF-*β*1 and NOX4 expression in the LGGs and GBMs was analyzed with TCGA database. Data represent mean and SD of three independent experiments. ns: not significant; ^∗^*P* < 0.05; ^∗∗^*P* < 0.01; ^∗∗∗^*P* < 0.001.

To determine the role of TGF-*β*1 and NOX4 in the development of glioblastoma, the expression of TGF-*β*1 and NOX4 was analyzed in normal brain, LGG, and GBM tissues. Compared to that in the normal brain and LGG tissues, TGF-*β*1 and NOX4 mRNA expression were significantly upregulated in the GBM tissues in TCGA database (Figure 1(a)). Besides, by applying qPCR (Figure 1(b)), western blot (Figure 1(c), Fig. S1(a)), and immunochemistry (Figure 1(d)) on samples with different pathologic grades obtained from patients in Shandong Provincial Hospital, we found that compared with normal brain and LGG tissues, TGF-*β*1 and NOX4 mRNA and protein expression levels in GBM tissues were significantly higher. Thus, the high expression levels of TGF-*β*1 and NOX4 are associated with a higher grade of human glioma. Furthermore, TGF-*β*1 and NOX4 expression levels were also related to the prognosis of glioma patients in TCGA database. We found that the LGG and GBM patients with high TGF-*β*1 or NOX4 expression had a worse prognosis than patients with low TGF-*β*1 or NOX4 expression in TCGA database (Figures 1(e) and 1(f)). In summary, these results strongly suggest that TGF-*β*1 and NOX4 might serve as prognostic biomarkers in glioma.

### 3.2. TGF-*β*1 Induces NOX4 and ROS via Smad Signal Pathway in Glioblastoma

**Figure 2:**
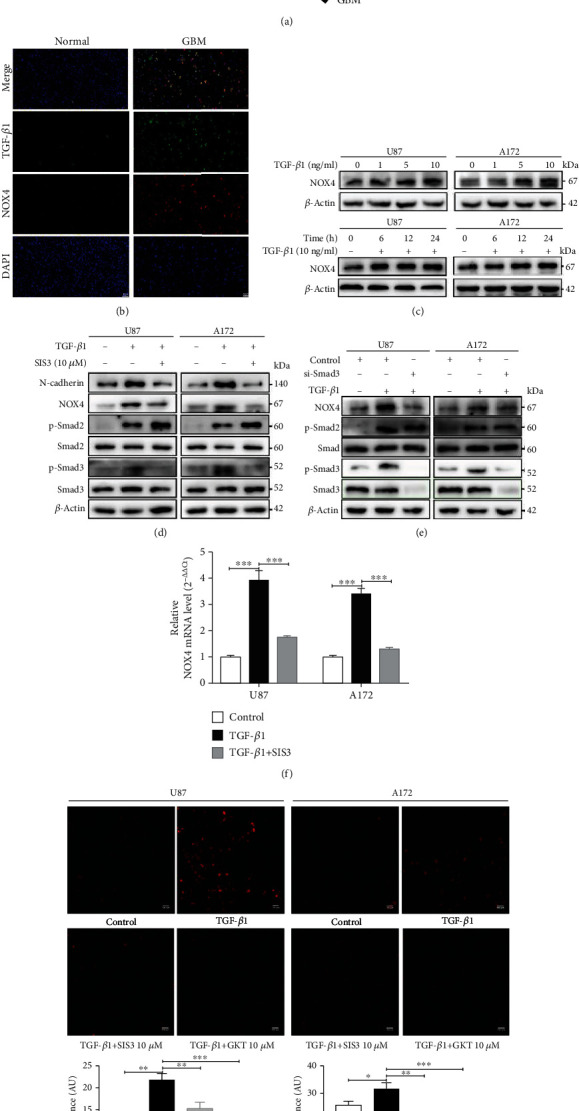
TGF-*β*1 induces NOX4 and ROS via Smad signal pathway in glioblastoma. (a) The correlation between TGF-*β*1 expression and NOX4 expression in glioma patients according to TCGA database and clinical specimens. (b) Immunofluorescent analysis of TGF-*β*1 (green) and NOX4 (red) coexpression in normal brain samples and GBM tissues. Scale bar = 50 *μ*m. (c) Western blot analysis of the NOX4 protein expression levels with different concentrations of TGF-*β*1 (0, 1, 5, and 10 ng/ml) after 24 hours and time-dependent effects of TGF-*β*1 (10 ng/ml) treatment as evaluated for the glioblastoma cells. (d) Western blot analysis of the protein levels of N-cadherin, NOX4, Smad3, p-Smad3, Smad2, and p-Samd2 in glioblastoma cells treated with TGF-*β*1 in the presence or absence of SIS3 (10 *μ*M) for 24 hours. (e) Western blot analysis for NOX4, p-Smad3, and p-Smad2 from the glioblastoma cells transfected with si-NC or si-Smad3 and then treated with TGF-*β*1 for 24 hours. (f) qPCR analysis of the NOX4 mRNA levels in glioblastoma cells treated with TGF-*β*1 in the presence or absence of SIS3 for 24 hours. (g) Glioblastoma cells were treated with TGF-*β*1 for 24 hours in the absence or presence of SIS3 (10 *μ*M) and GKT137831 (10 *μ*M) before staining for reactive oxygen species with CellROX Deep Red Reagents. ROS levels were calculated by the average fluorescent intensity. Scale bar = 100 *μ*m. Data represent mean and SD of three independent experiments. ^∗^*P* < 0.05; ^∗∗^*P* < 0.01; ^∗∗∗^*P* < 0.001.

The signaling pathway that regulates NOX4 expression in glioblastomas is critical in exploring treatments. NOX4 was regulated by the TGF-*β*1 signaling pathway in some tumors and tissues and then promoted malignant progress [11, 12]. There was a correlation between TGF-*β*1 and NOX4 expression in glioma patients in TCGA databases as well as in our tissue specimens (Figure 2(a)). To further explore the relationship between TGF-*β*1 and NOX4, the immunofluorescence images indicated that these two molecules were colocalized and coexpression in GBM tissues was compared with normal brain tissues (Figure 2(b)). Through western blot analysis, we observed that the protein expression of NOX4 was upregulated with increasing concentrations of TGF-*β*1 after 24 hours in glioblastoma cells (Figure 2(c), Fig. S2(a)). To determine the proper time required for TGF-*β*1 stimulation, we set a gradient time course and analyzed the results by western blot (Figure 2(c), Fig. S2(a)). Our results indicated that 10 ng/ml and 24 hours were appropriate to induce NOX4 expression. In contrast, the promoting effect of TGF-*β*1 on NOX4 expression was blocked by SIS3 (10 *μ*M), a specific inhibitor of Smad3 but not Smad2 in the TGF-*β*1/Smad pathway (Figure 2(d), Fig. S2(b)). Furthermore, to determine whether NOX4 was regulated by TGF-*β*1 through the Smad pathway, the glioblastoma cells were transfected with si-NC or si-Smad3. As expected, the protein levels of NOX4 were decreased in the Smad3-silenced group compared with the control group in the presence of TGF-*β*1 (Figure 2(e), Fig. S2(c)). We also further confirmed these results by qPCR that the mRNA levels of NOX4 were increased stimulated by TGF-*β*1 and revered in the presence of SIS3 (Figure 2(f)). Meanwhile, increased ROS levels were observed in the glioblastoma cells after treated with TGF-*β*1 (Figure 2(g)). ROS, which is a product of NAD(P) H oxidase, production has been recognized to have a critical role in a range of tumor processes in GBM [24]. Our data found that TGF-*β*1 could increase ROS levels in the human glioblastoma cells and was inhibited by the Smad3 inhibitor SIS3 and the NOX4 selective inhibitor GKT137831 (10 *μ*M) (Figure 2(g)). But western blot of NOX4 showed no change of GKT137831 treatment supports a view that GKT137831 inhibits NOX4 function directly and not indirectly via an effect on NOX4 expression or stability (Fig. S2(d)). Furthermore, we evaluated the subcellular localization of NOX4, and the results suggested that NOX4 localized to the cytoplasm but not only confined to mitochondria in glioblastoma cells (Fig. S2(e)). Thus, the above results indicate that TGF-*β*1 induces NOX4 accompanied by ROS via the Smad pathway in glioblastoma and suggest NOX4 is one of the major sources of ROS produced in the presence of TGF-*β*1.

### 3.3. NOX4-Derived ROS Mediates TGF-*β*1-Induced Metabolic Reprogramming in Glioblastoma Cells

**Figure 3:**
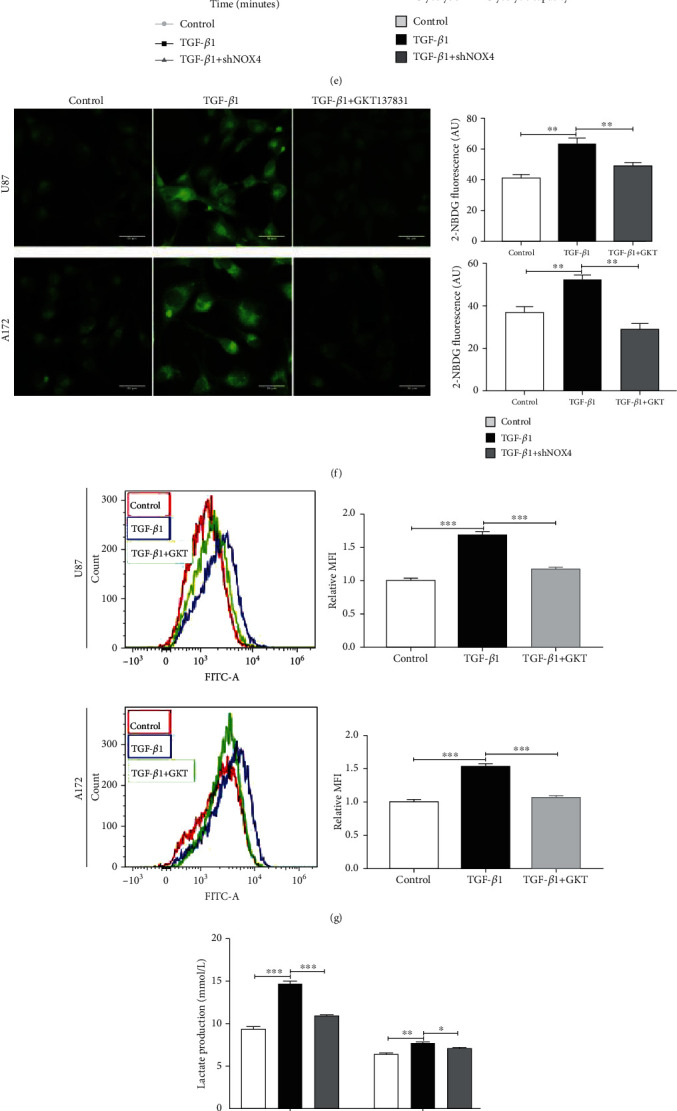
NOX4-derived ROS mediates TGF-*β*1-induced metabolic reprogramming in glioblastoma cells. (a) Western blot analysis showed the time-dependent effects of metabolic enzymes under TGF-*β*1 treatment (10 ng/ml) on glioblastoma cells. (b) ROS generation was examined in normal glioblastoma cells transfected with sh-NC or shNOX4. Scale bar = 100 *μ*m. (c) Western blot analysis for metabolic enzymes when NOX4 was suppressed or knocked down under TGF-*β*1 treatment on glioblastoma cells. (d) qPCR was conducted in control and shNOX4 glioblastoma cells to determine the mRNA levels of metabolic enzymes treated with TGF-*β*1. (e) Glioblastoma cells were treated with TGF-*β*1 for 24 hours before being adhered to microplates, and extracellular acidification rate (ECAR) was determined over time and analyzed as bar graphs. (f) Immunofluorescence images and (g) flow cytometry results of glucose uptake in glioblastoma cells determined by staining with 2-NBDG after treated with TGF-*β*1 in the absence or presence of GKT137831. Glucose uptake ability was calculated by the average fluorescent intensity. Scale bar = 50 *μ*m. (h) Lactate production was determined in glioblastoma cells with or without NOX4 knockdown under treatment of TGF-*β*1 for 24 hours. (i) Glioblastoma cells were treated with TGF-*β*1 for 24 hours before being adhered to microplates, and oxygen consumption rate (OCR) was determined over time and analyzed as bar graphs. (j) Immunofluorescence microscopy of mitochondria in glioblastoma cells transfected with sh-NC or shNOX4 stimulated by TGF-*β*1 for 24 hours. The mitochondrial mass was visualized by MitoTracker staining and normalized by fluorescence. Scale bar = 50 *μ*m. (k) Glioblastoma cells with or without NOX4 knockdown were treated with TGF-*β*1 for 24 hours before undergoing a PDH activity assay and western blot for the analysis of phosphorylated PDH. Data represent mean and SD of three independent experiments. ^∗^*P* < 0.05; ^∗∗^*P* < 0.01; ^∗∗∗^*P* < 0.001.

To determine the effects of TGF-*β*1 on metabolic enzyme expression, we performed a western blot in glioblastoma cells after treatment with TGF-*β*1. TGF-*β*1 increased protein expression levels of glucose transporter 1 (GLUT1), hexokinase-2 (HK2), lactate dehydrogenase-A (LDHA) in a time-dependent manner, which are closely related to glycolysis, and pyruvate dehydrogenase kinase-1 (PDK1), an important enzyme related to mitochondrial respiration, also increased (Figure 3(a), Fig. S3(a)). In contrast, the protein expression of GLUT1, HK2, LDHA, and PDK1 has blocked as well as ROS generation when NOX4 was knocked down by NOX4-specific shRNA lentivirus or inhibitor (Figures 3(b) and 3(c), Fig. S3(b), (c)). Similar results were obtained in the qPCR analysis (Figure 3(d)). Based on the treatment of TGF-*β*1, it dramatically increased cellular ROS levels, whereas NOX4 inhibition reversed this phenotype, and we found that combinatorial treatment of TGF-*β*1 with ROS scavenger N-acetyl-cysteine (NAC, 5 mM) restored TGF-*β*1-induced metabolic enzyme expression (Fig. S3(d)). To functionally characterize whether NOX4 is required for TGF-*β*1-induced metabolic phenotype, glioblastoma cells were subjected to a glycolysis stress test and a mitochondrial stress test following 24 hours of treatment with TGF-*β*1. We added glucose, oligomycin A, and 2-DG to transduce glioblastoma cells then determine their extracellular acidification rate (ECAR, an indicator of glycolytic lactate production) using an extracellular flux analyzer. The results showed that TGF-*β*1 increased both the basal glycolysis and glycolytic capacity. Strikingly, NOX4 knockdown substantially reduced the basal glycolysis as well as glycolytic capacity compared with control cells under treatment of TGF-*β*1 (Figure 3(e)). Glucose uptake assays were also used to identify the metabolic phenotypes of glioblastoma cells. TGF-*β*1 treatment increased glucose uptake as evidenced by increased 2-NBDG fluorescence and at the same time inhibited NOX4 with GKT137831 which decreased the 2-NBDG uptake (Figure 3(f)). With the 2-NBDG fluorescence staining detection on the flow cytometer, we can gain a similar result (Figure 3(g)). Elevated lactic acid production also is a feature of the Warburg effect. Consistently, lactate production was increased after TGF-*β*1 treatment and decreased when NOX4 was knocked down (Figure 3(h)). Nevertheless, oxygen consumption rate (OCR), estimated using a methodology similar to that used for ECAR measurement, was lower when treated with TGF-*β*1 compared to the control cells. NOX4 knockdown reversed the TGF-*β*1-induced reduction in OCR (Figure 3(i)). Mitochondria mass increased when mitochondrial dysfunction occurred [25]. Therefore, we introduced the MitoTracker Red probe to observe the mitochondria mass and localization. As is expected, TGF-*β*1 significantly increased the mitochondria mass, and silencing NOX4 reduced the mitochondria mass in TGF-*β*1-stimulated cells (Figure 3(j)). Pyruvate dehydrogenase (PDH), which is inhibited by PDK1, can directly convert pyruvate into acetyl-CoA. To determine whether the TGF-*β*1/NOX4 signaling-induced increase in PDK1 affects PDH function, we measured PDH activity and phosphorylation of PDH. TGF-*β*1 decreased PDH activity and phosphorylated PDH while NOX4 knockdown reversed these phenotypes (Figure 3(k), Fig. S3(e)). Together, these results demonstrate that NOX4 is required for TGF-*β*1 alters metabolic reprogramming, namely, induces glycolysis and reduces mitochondrial respiratory capacity.

### 3.4. TGF-*β*1 Induces Epithelial-to-Mesenchymal Transition and Proliferation, Migration, and Invasion via NOX4/ROS Signal Pathway of Glioblastoma Cells

**Figure 4:**
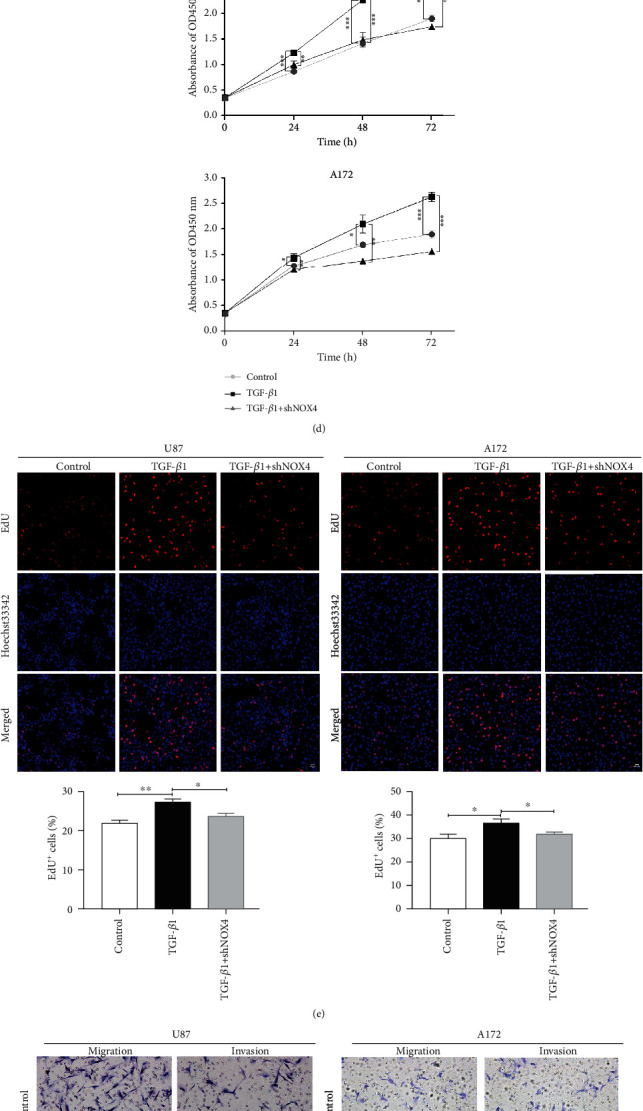
TGF-*β*1 induces epithelial-to-mesenchymal transition and proliferation, migration, and invasion via NOX4/ROS signal pathway of glioblastoma cells. (a) Western blot analysis showed the time-dependent effects of EMT markers under TGF-*β*1 treatment (10 ng/ml) on glioblastoma cells. (b) Western blot analysis of EMT markers when NOX4 was suppressed and deleted under TGF-*β*1 treatment (10 ng/ml) for 24 hours on glioblastoma cells. (c) Immunofluorescences observed Vimentin expression in glioblastoma cells after treated with TGF-*β*1. Scale bars = 20 *μ*m. (d) CCK-8 assays were performed to evaluate the glioblastoma cell's viability and growth. (e) EdU proliferation assays were performed to evaluate the growth of glioblastoma cells. The EdU incorporation was quantitated. Scale bar = 50 *μ*m. (f) Crystal violet stained sections of the Matrigel matrix. Migration and invasion ability of glioblastoma cells were determined by transwell assay. The average number of migrating and invading cells was counted after 24 hours of migration and invasion. Scale bars = 100 *μ*m. Data represent mean and SD of three independent experiments. ^∗^*P* < 0.05; ^∗∗^*P* < 0.01; ^∗∗∗^*P* < 0.001.

Excess ROS production has been implicated in EMT gene regulation, cell proliferation, and migration [21]. It is widely accepted that EMT plays an important role in the invasion and metastasis of different tumors [26]. To determine whether TGF-*β*1 induced changes in EMT gene expression in glioblastoma cells, we measured the expression of EMT makers treated with TGF-*β*1. First, TGF-*β*1 treated upstream mesenchymal subtype markers (N-cadherin and Vimentin) and decrease epithelial marker (E-cadherin) compared to the control cells required time-dependent (Figure 4(a), Fig. S4(a)). We found that 24 hours was the appropriate time for TGF-*β*1 inducing EMT which corresponds to the time for the metabolic enzyme. Besides, NOX4 silencing or inhibiting caused a significant increase in the epithelial markers and a decrease in mesenchymal subtype marker treated with TGF-*β*1 (Figure 4(b), Fig. S4(b)). Immunofluorescence also demonstrated the same change of Vimentin between control and shNOX4 glioblastoma cells after treatment with TGF-*β*1 for 24 hours (Figure 4(c)). To determine if NOX4-derived ROS is involved in TGF-*β*1-mediated proliferation of glioblastoma cells, we performed a CCK-8 assay on glioblastoma cells and examined the effects of NOX4-NC or NOX4-shRNA in the presence of TGF-*β*1. The results of the CCK-8 assays showed that when treated with TGF-*β*1, it promoted cell proliferation, while downregulation of NOX4 suppressed the proliferation of glioblastoma cells (Figure 4(d)). To further confirm that TGF-*β*1 directly stimulated GBM cells' proliferation, we treated glioblastoma cells with TGF-*β*1 and determined the cell growth by EdU proliferation assays. In agreement with the CCK-8 assays, EdU proliferation assays showed that the growth of the cells was apparently accelerated in the presence of TGF-*β*1. Besides, NOX4 silencing significantly decreased the percentage of positive cells compared with the control cells, in the presence of TGF-*β*1 (Figure 4(e)). All these data collectively indicated that NOX4 mediated TGF-*β*1-induced GBM cell proliferation. Cells' invasion and migration were investigated by transwell invasion and migration assays with or without Matrigel. The results showed that the numbers of crystal violet staining positive cells in the TGF-*β*1-treated groups were significantly increased compared with the corresponding control group while NOX4 silencing reversed the effect (Figure 4(f)). These data suggested that NOX4 simultaneously mediated TGF-*β*1-induced glioblastoma cells' migration and invasion. Together, our results indicate that NOX4/ROS driven by TGF-*β*1 mediates tumorigenesis in glioblastoma cells.

### 3.5. Metabolic Reprogramming Induced by TGF-*β*1/NOX4/ROS Axis Is Required for Epithelial-to-Mesenchymal Transition

**Figure 5:**
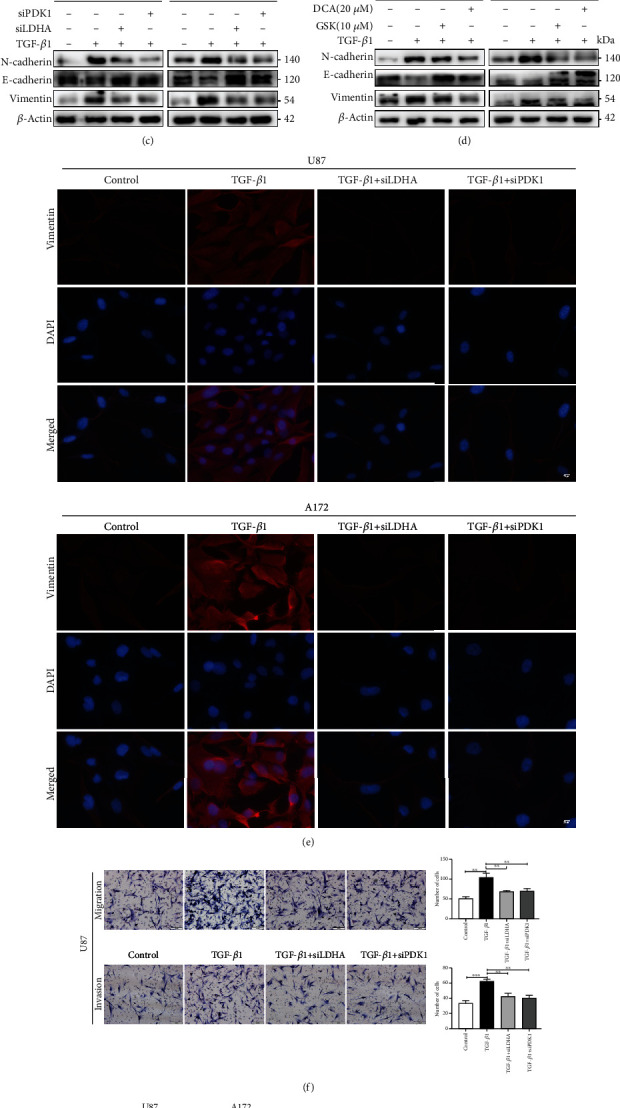
Metabolic reprogramming induced by TGF-*β*1/NOX4/ROS axis is required for epithelial-to-mesenchymal transition. (a) Glioblastoma cells transfected with siRNA control and siLDHA and U87 cells were treated with TGF-*β*1 for 24 hours before being adhered to microplates, and extracellular acidification rate (ECAR) was determined over time and analyzed as bar graphs. (b) Glioblastoma cells transfected with siRNA control and siPDK1 and U87 cells were treated with TGF-*β*1 for 24 hours before being adhered to microplates, and oxygen consumption rate (OCR) was determined over time and analyzed as bar graphs. (c) EMT protein marker expression in glioblastoma cells treated with the siLDHA and siPDK1 in the presence of TGF-*β*1 for 24 hours. (d) EMT protein marker expression in glioblastoma cells when treated with LDHA inhibitor GSK2837808A (10 *μ*M) and PDK1 inhibitor Dichloroacetate (20 *μ*M) combined with TGF-*β*1 for 24 hours. (e) Immunofluorescences observed Vimentin expression in glioblastoma cells after treated with siLDHA and siPDK1 under treatment of TGF-*β*1. Scale bars = 20 *μ*m. (f) Migration and invasion ability of glioblastoma cells transfected with siLDHA and siPDK1 were determined by transwell assay under treatment of TGF-*β*1. Scale bars = 100 *μ*m. (g) Glioblastoma cells were treated with rotenone (50 nM) for 24 hours before staining for reactive oxygen species with CellROX Deep Red Reagents. Scale bars = 100 *μ*m. (h) EMT protein marker expression when inhibited mitochondrial respiration with rotenone (50 nM) for 24 hours in glioblastoma cells. Data represent mean and SD of three independent experiments. ^∗^*P* < 0.05; ^∗∗^*P* < 0.01; ^∗∗∗^*P* < 0.001.

We then investigated whether modulation of metabolism influences epithelial-to-mesenchymal transition, migration, and invasion in glioblastoma cells. To inhibit TGF-*β*1-induced glycolysis, we treated glioblastoma cells with siRNA against LDHA (siLDHA), which was the most highly upregulated glycolytic gene under treatment with TGF-*β*1. LDHA knockdown cells had reduced LDHA protein levels and exhibited reduced ECAR when compared with control knockdown cells (Figure 5(a)). Inhibition of LDHA (or glycolysis) reduced TGF-*β*1-induced N-cadherin and Vimentin protein expression meanwhile increased E-cadherin protein expression (Figures 5(c) and 5(e), Fig. S5(a)). To rescue the TGF-*β*1-induced reduction in mitochondrial oxidative phosphorylation, we treated glioblastoma cells with siRNA against PDK1, a mitochondrial respiration-related gene that was overexpressed under the action of TGF-*β*1. PDK1 knockdown cells had reduced PDK1 protein levels, and genetic inhibition of PDK1 expression by siRNA partially improved maximal respiration and respiratory capacity induced by TGF-*β*1 (Figure 5(b)). Inhibition of PDK1 also significantly reduced N-cadherin and Vimentin gene expression meanwhile increased E-cadherin gene expression (Figures 5(c) and 5(e), Fig. S5(a)). Similarly, glioblastoma cells treated with GSK2837808A (GSK, 10 *μ*M), a selective inhibitor of LDHA and Dichloroacetate (DCA, 20 *μ*M), a selective inhibitor of PDK1, reversed the EMT protein markers induced by TGF-*β*1 (Figure 5(d)). Besides, genetic inhibition of LDHA and PDK1 reversed the TGF-*β*1-induced glioblastoma cells' migration and invasion (Figure 5(f)). Mitochondrial dysfunction induces EMT, cell migration, and invasion in some types of tumor cells [27]. To further explore whether the inhibition of oxidative phosphorylation by itself would further increase EMT in glioblastoma cells, we treated our cells with rotenone, an inhibitor of the mitochondrial respiratory complex which blocks electron flow in the mitochondria and results in ROS overproduction [28]. We found that inhibition of the electron transport chain with a low dose of rotenone (50 nM) increased ROS production (Figure 5(g)) as well as downregulated expression of the epithelial protein marker E-cadherin and upregulated expression of the mesenchymal protein markers Vimentin and N-cadherin (Figure 5(h), Fig. S5(b)) which also confirmed that mitochondrial dysfunction and ROS production were necessary for EMT process in glioblastoma cells. These results are consistent with block NOX4, LDHA, and PDK1 reducing glycolysis and improving mitochondrial capacity, as well as reducing EMT, undertreated with TGF-*β*1. In summary, these findings suggest that TGF-*β*1-induced metabolic reprogramming, namely, induction of glycolysis and inhibition of mitochondrial oxidation, is required for the EMT phenotype.

### 3.6. PI3K/AKT/HIF-1*α* Signal Pathways Involve in the Metabolic Reprogramming and EMT Induced by TGF-*β*1/NOX4/ROS Axis

**Figure 6:**
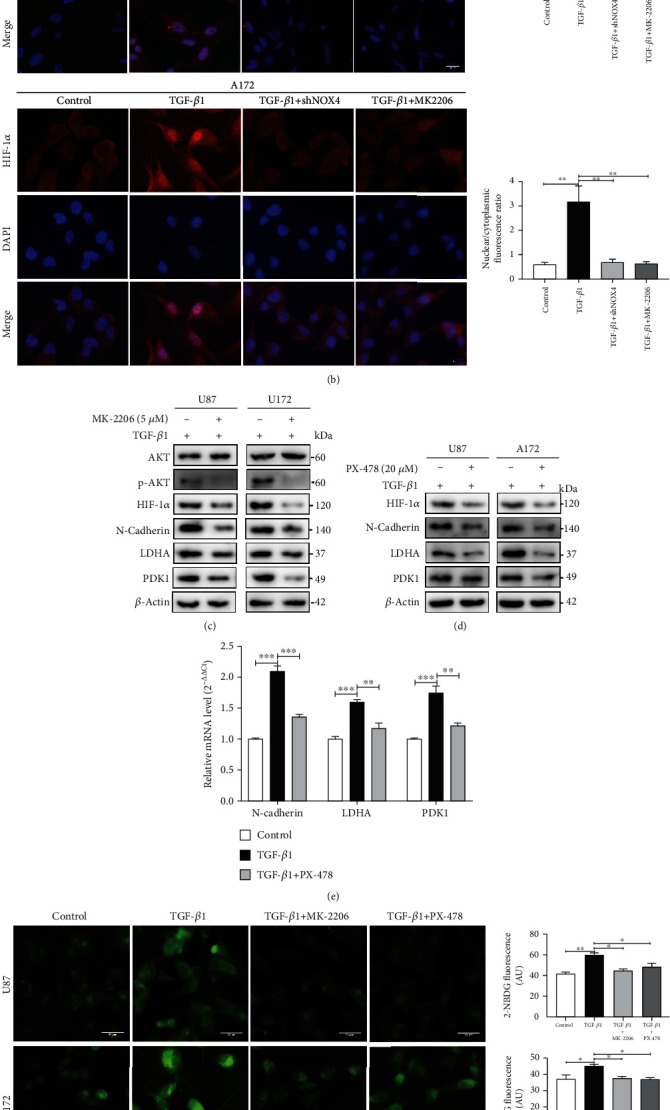
PI3K/AKT/HIF-1*α* signal pathways involve in the metabolic reprogramming and EMT induced by TGF-*β*1/NOX4/ROS axis. (a) The activity or expression of PI3K, AKT, p-AKT, and HIF-1*α* was detected by western blot. (b) Immunofluorescences observed HIF-1*α* expression in glioblastoma cells in the presence or absence of TGF-*β*1 and MK-2206 (5 *μ*M) for 24 hours. Nuclear localization of HIF-1*α* was expressed as the ratio of nuclear to cytoplasmic fluorescence. Scale bars = 50 *μ*m. (c) The activity or expression of indicated proteins was detected by western blot when p-AKT was inhibited by the MK-2206 in the TGF-*β*1-treated glioblastoma cells. (d) The activity or expression of indicated proteins was detected by western blot when HIF-1*α* was inhibited by treatment with PX-478 (20 *μ*M) for 24 hours in TGF-*β*1-treated cells. (e) qPCR was conducted in U87 cells to determine the mRNA levels of N-cadherin, LDHA, and PDK1 treated without and with TGF-*β*1 or TGF-*β*1plus PX-478. (f) Immunofluorescence images of glucose uptake in glioblastoma cells determined by staining with 2-NBDG when AKT was inhibited by the MK-2206 and HIF-1*α* were inhibited by PX-478 in the TGF-*β*1-treated cells. Scale bar = 50 *μ*m. (g) Immunofluorescence microscopy of mitochondria in glioblastoma cells stimulated by TGF-*β*1 treated with MK-2206 and PX-478. The mitochondrial mass was visualized by MitoTracker staining. Scale bar = 50 *μ*m. Data represent mean and SD of three independent experiments. ^∗^*P* < 0.05; ^∗∗^*P* < 0.01; ^∗∗∗^*P* < 0.001.

PI3K/AKT has been reported to be an important regulator of EMT and metabolic reprogramming induced by TGF-*β*1 [29, 30]. HIF-1*α*, which is induced by growth factors, hypoxia, and oncogenes, plays a critical role in tumor metabolic reprogramming, growth, and angiogenesis [31–33]. Thereby, we hypothesized that TGF-*β*1-induced NOX4/ROS might play a tumorigenic role through activation of the PI3K/AKT/HIF-1*α* pathway. To prove our hypothesis, we explored the changes in the PI3K/AKT/HIF-1*α* pathway. Western blot analysis indicated that TGF-*β*1 treating induced marked activation of PI3K, phosphorylated AKT, and upregulated HIF-1*α*. NOX4 knockdown in our glioblastoma cells attenuated the activity of PI3K, p-AKT, and HIF-1*α* under treatment with TGF-*β*1 (Figure 6(a), Fig. S6(a)). To further verify the role of HIF-1*α* in the TGF-*β*1/NOX4/ROS axis, immunofluorescence was used to observe the distribution of HIF-1*α*. As expected, TGF-*β*1 induced HIF-1*α* overexpression and nuclear accumulation, which might strengthen its activity in glioblastoma cells (Figure 6(b)). Besides, NOX4 knockdown reduced the expression and nuclear accumulation of HIF-1*α* (Figure 6(b)). Then, the glioblastoma cells were treated with TGF-*β*1 plus MK-2206 (5 *μ*M), a highly selective inhibitor of AKT, for 24 hours. Our results indicated that the expression and nuclear accumulation of HIF-1*α* were also significantly reduced in TGF-*β*1-induced cells treated with MK-2206 (Figure 6(b)). Moreover, AKT inhibitor MK-2206 and HIF-1*α* inhibitor PX-478 (20 *μ*M) significantly reduced the protein expression of HIF-1*α*, N-cadherin, LADH, and PDK1 (Figures 6(c) and 6(d), Fig. S6(b), (c)). qPCR analysis was used to confirm the effect of nuclear accumulation for HIF-1*α*. Our results revealed that TGF-*β*1 could increase the expression of N-cadherin, LDHA, and PDK1 mRNA expression whereas HIF-1*α* inhibitor could reverse the effect of HIF-1*α* nuclear accumulation (Figure 6(e)). To better understand the role of HIF-1*α* regulating related gene expression under the treatment of TGF-*β*1, glioblastoma cells were transfected with si-NC or si-HIF-1*α*. Consistently, we found that si-HIF-1*α* successfully knocked down HIF-1*α* expression as well as reversed the TGF-*β*1-induced increase in N-cadherin, LDHA, and PDK1 levels (Fig. S6(d)). However, the change of mRNA levels of HIF-1*α* was not obvious treated with TGF-*β*1 or MK-2206 in glioblastoma cells (Fig. S6(e)). Subsequently, we evaluated the impact of AKT and HIF-1*α* on NOX4. Inhibiting AKT with MK-2206 or HIF-1*α* with PX-478 reduced the protein level of NOX4 (Fig. S6(f)) which indicated that a potential positive feedback loop between NOX4 and HIF-1*α*. In addition, by either protein inhibited of AKT and HIF-1*α*, we provided direct evidence that the PI3K/AKT/HIF-1*α* pathway contributed to TGF-*β*1 mediated glucose uptake and mitochondrial dysfunction (Figures 6(g) and 6(h)). Besides, AKT and HIF-1*α* were required for TGF-*β*1-induced increase in glycolysis and reduction in mitochondrial respiration (Fig. S6(g)). Collectively, our results demonstrate that PI3K/AKT/HIF-1*α* signal pathways involve in the metabolic reprogramming were induced by TGF-*β*1/NOX4/ROS axis during EMT.

### 3.7. Inhibiting NOX4 Suppresses Tumorigenesis Stimulated by TGF-*β*1 in Xenograft Mice

**Figure 7:**
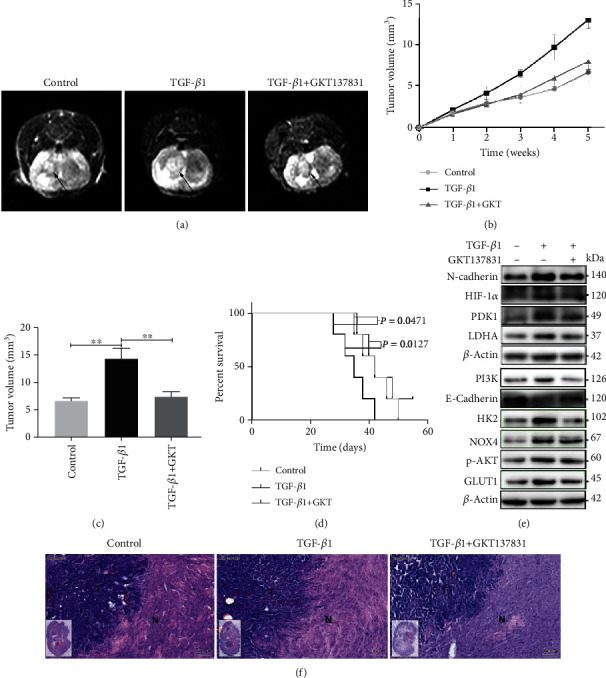
Inhibition of NOX4 suppressed tumorigenesis in xenograft mice. (a) The representative image of intracranial tumors of mice was monitored and measured by MRI. (b) Tumor volume monitored by MRI. (c) The tumor volume was measured by MRI. (d) The percentage of the number of mice remaining after they received intracranial injections of either control or TGF-*β*1 alone or GKT137831 in combination at the indicated doses. (e) Western blot for the indicated proteins of intracranial tumors from the mice. (f) HE staining of control, TGF-*β*, and TGF-*β*/GKT137831 groups, and the tumor borders as well as infiltrating and invasion of the tumor are shown (N: normal brain tissue; T: tumors in brain; scale bar = 100 *μ*m). Data represent mean and SD of three independent experiments. ^∗^*P* < 0.05; ^∗∗^*P* < 0.01; ^∗∗∗^*P* < 0.001.

Finally, we investigated the function of NOX4 regulates the tumorigenesis of glioblastoma cells with intranasal TGF-*β*1 administration *in vivo*. Intranasal administration of TGF-*β*1 can be transported into the central nervous system and subsequently exert its biological effects by regulating gene expressions of TGF-*β* receptors [34]. After the U87 cells were intracranially implanted into immunocompromised mice, we assigned the animals to the following treatment groups: PBS group (control group), TGF-*β*1 group, and TGF-*β*1 plus GKT137831 group. Tumor volume results monitored and measured by MRI showed that intranasal administration of TGF-*β*1 significantly increased the volume of tumors while NOX4 suppressed with GKT137831 undertreated with TGF-*β*1 administration reduced tumor volume compared with TGF-*β*1 administration alone (Figures 7(a)–7(c)). The overall survival was also higher in the TGF-*β*1 plus GKT137831 group than in the TGF-*β*1 group (Figure 7(d)). Western blot of xenograft tumors showed that intranasal administration of TGF-*β*1 significantly increased the expression of N-cadherin, PI3K, p-AKT, HIF-1*α*, NOX4, and metabolic enzymes HK2, GLUT1, LDHA, and PDK1; meanwhile, NOX4 suppressed and reduced the levels of that protein compared with the TGF-*β*1 group (Figure 7(e), Fig. S7(a)). HE staining of the tumors showed that TGF-*β*1 plus GKT137831 group demonstrated that the edge of the tumor was clearer as well as less tumor-infiltrating and invasion than the TGF-*β*1 group (Figure 7(f)). Thus, these results show that NOX4 mediates intranasal TGF-*β*1 treatment-induced growth, invasion, metabolic reprogramming, and EMT *in vivo*.

## 4. Discussion

In the present study, we investigated one finding that TGF-*β*1 and NOX4 are highly expressed in GBM tissues compared with LGG or normal brain tissues and correlated with each other in TCGA database and clinical samples. Besides, TGF-*β*1 significantly upregulates the expression of NOX4 mRNA and protein levels through Smad signaling pathway in glioblastoma cells. Furthermore, we found that TGF-*β*1 increases glycolysis and reduces mitochondrial respiratory capacity by promoting NOX4-dependent ROS-mediated HIF-1*α* nuclear accumulation and stabilization in glioblastoma cells. Additionally, another major finding of the present study is the demonstration that TGF-*β*1-induced metabolic reprogramming contributes to EMT, migration, and invasion.

TGF-*β* is known as multifunctional cytokine growth factors that participate in the regulation of key events of cell proliferation, survival, differentiation, and involvement in the creation of a protumorigenic microenvironment. In the microenvironment of glioblastoma, microglial cells and macrophages accumulate in and around glioblastoma tissue and synthesize and release TGF-*β* which actively promotes glioblastoma growth and invasion [35]. Therefore, we utilized recombinant TGF-*β*1 to simulate a special tumorigenic microenvironment of glioblastoma and detected the effects of TGF-*β*1 pretreatment on regulating cell properties under EMT conditions *in vitro* and *in vivo* in the present study. Indeed, TGF-*β*1 can regulate brain tumor progression [36]. Previous reports indicate TGF-*β*1 can modulate glycolysis of glioblastoma cells through directly regulating metabolic enzyme expression [29]. Our results demonstrated that TGF-*β*1 increases metabolic associated enzymes in a time- and dose-dependent fashion and confirmed 24 hours and 10 ng/ml are appropriate to induce glioblastoma metabolic changes. These conditions are highly consistent with TGF-*β*1-induced increasing EMT gene expression which corresponds with the Warburg effect and explains why cancer cells towards increasing glycolytic metabolism even in the presence of abundant oxygen despiting it being an inefficient way to generate ATP. We speculate that TGF-*β*1-induced metabolic reprogramming gives glioblastoma cells a growth advantage by providing energy and essential cellular ingredients for cell growth. Consist with this hypothesis, increasing glycolysis and reducing respiration under treatment TGF-*β*1 result in EMT in our results. Mitochondria are well known for their role as cellular powerhouses, which provide large amounts of ATP for the cell, and their dysfunction was associated with the various biological process including metabolism and epithelial-to-mesenchymal transition [37]. TGF-*β*1 induces mitochondria dysfunction in fibroblasts and macrophages cells [25, 38]; however, few studies to date have investigated the effect of TGF-*β*1 on mitochondria dysfunction in glioblastoma cells. As we showed in the results, TGF-*β*1 inhibited mitochondrial respiration and increased the mitochondrial mass in the meantime leading to changes in the expression of EMT genes.

Recent research shows that targeted TGF-*β* signaling in CD4+ T cells revives helper T cell responses and suppresses cancer progression, and pharmacological inhibition of TGF-*β* signaling in helper T cells may define a novel immunotherapy approach targeting the cancer environment in cell and mouse models [39]. However, systemic treatment with TGF-*β* inhibitors comes with side effects due to the pleiotropic effect of the pathway and, therefore, is unacceptable or challenging for utilizing within the clinic [40]. In turn, it is necessary to find the exact tumor supporting downstream impacts of TGF-*β* signaling to supply new opportunities for targeting. In our study, we put NOX4-dependent ROS-mediated metabolic reprogramming in the spotlight. NOX4 but not other Nox homologues is abundantly expressed in glioblastoma cells, and its elevated expression NOX4 appears to be involved in cell proliferation and survival and associates with poor clinical outcomes [13]. A recent study reported that inhibition of NOX4 appears to be a potential anticancer strategy of GBM [41]. However, the underlying molecular mechanisms of why NOX4 is elevated in glioblastoma are still poorly understood. Our results demonstrated that NOX4 accompanied by ROS was induced by TGF-*β*1 in a time- and dose-dependent fashion through Smad-dependent pathway. Non-Smad signaling pathways such as the p38 and Jun N-terminal kinase (JNK) mitogen-activated protein kinase (MAPK) pathways can also be initiated downstream of TGF-*β* signaling [42]. Thus, further studies are still needed to elucidate the detailed mechanisms that whether TGF-*β*1 induced NOX4 as well as ROS through non-Smad pathways in glioblastoma cells.

NADPH oxidases (NOXs) and mitochondria are two main providers of endogenous ROS in tumor cells [21]. In this study, TGF-*β*1-mediated ROS was significantly reduced when NOX4 was suppressed indicating NOX4 is the main source of ROS generation rather than mitochondria stimulated by TGF-*β*1. Increasingly, ROS production contributes to the biological processes of cancer. In tumor cells, toxic levels of ROS production can activate antitumorigenic signaling resulting in oxidative stress-induced cancer cell death [43]. In contrast, several reports have shown that a high level of ROS, which is generated either during respiration or in a targeted manner by a class of NADPH oxidases, maintains protumorigenic signaling, resulting in a loss of tumor suppressor gene function, adaptations to hypoxia, increased glucose metabolism, and resistance to apoptosis attribute to strives to scavenge excessive ROS [21]. Our results are consistent with the evidence, and we found that TGF-*β*1 induced NOX4-generated moderate ROS in glioblastoma cells and then promotes glycolysis and inhibits mitochondrial respiration simultaneously. In addition, TGF-*β*1-driven ROS production and metabolic reprogramming are reversed when NOX4 expression is depleted or inhibited. Another data support ROS played a pivotal role in TGF-*β*1-mediated glioblastoma cell metabolic reprogramming is that the scavenging of total ROS using NAC significantly abolished the metabolic enzymes induced by TGF-*β*1. In summary, we conclude that NOX4 acts as a downstream molecule of TGF-*β*1, and its overexpression results in ROS accumulation contribute to metabolic reprogramming and EMT. Thereby, intracellular ROS, especially mediated by NOX4, is a potential therapeutic strategy for treating glioblastoma.

Reprogramming of energy metabolism is a hallmark of cancer during multistep development and contributes to disease progression and tumorigenesis [3]. EMT and metabolism are intertwined, and understanding the supporting molecular determinants of this relation will reveal new insights into the formation and dissemination of tumors and potentially provide novel therapeutics to specifically treat cancer cells. In this study, we clearly demonstrated that the metabolic reprogramming is required for EMT within the brain tumor microenvironment. Other research has elucidated that enhanced glycolysis and disordered mitochondrial metabolism are associated with tumor invasion and EMT [37, 44], but no one has explored the relationship among TGF-*β*1 released from TME, metabolism, and EMT. Our study provides evidence that a temporary change in glucose metabolism is enough to inhibit the EMT of glioblastoma cells in a special tumorigenic microenvironment. Through suppressing the key of enzymes of glycolysis LDHA and mitochondrial metabolism PDK1, induced by TGF-*β*1, the mesenchymal subtype markers N-cadherin and Vimentin are negated, suggesting that the metabolic reprogramming is directly related to cancer metastasis. Consistent with TGF-*β*1-induced mitochondrial dysfunction associated with tumor invasion and metastasis, rotenone treatment disrupted the integrity of the respiratory chain, leading to ROS overproduction and drove glioblastoma cells to undergo EMT. These results give a molecular basis for why the glioblastoma cells adapt to aerobic glycolysis, due to enabling tumor cells to utilize glucose-derived carbons for the synthesis of essential cellular ingredients and grow into a more aggressive form.

The finding that incubation of TGF-*β*1 resulting in NOX4 overexpression caused significant stimulation of PI3K/AKT signaling in glioblastoma cells inspired us to investigate the connection between PI3K/AKT promotion and the tumorigenic effect of NOX4. The previous investigator demonstrated that ROS regulates biological function dependent on the PI3K/AKT signaling [45]. Likewise, we found elevated ROS generated by NOX4 under TGF-*β*1 activated the PI3K/AKT pathway, and NOX4 inhibition negatively regulated the PI3K/AKT pathway in glioblastoma cells. These data suggested that PI3K/AKT is a downstream pathway of NOX4-derived ROS. It is important to note that phosphorylation of AKT may lead to upregulation of HIF-1*α* expression and translocate to the nucleus in cancer cells [33]. HIF-1*α* can be transferred into the nucleus or accumulated in the nucleus after activation of the PI3K/AKT/mTOR pathway, and once it enters into the nucleus, this molecule can bind to related elements and thus promote the expression of genes involved in a variety of biological functions, such as cell proliferation, angiogenesis, migration, invasion, and metabolic reprogramming [33, 46, 47]. Decreased intracellular ROS suppressed the PI3K/AKT/HIF-1*α* signaling axis and then inhibited the glycolytic phenotype, cell proliferation, and migration invasion and induced cell apoptosis [21]. Based on these findings, we hypothesized that TGF-*β*1 treatment increases HIF-1*α* expression by stimulating the PI3K/AKT pathway in glioblastoma cell lines. We elucidated that overexpression of NOX4 induced by TGF-*β*1 can dramatically upregulate the phosphorylation of key components in PI3K/AKT signaling and subsequently induce HIF-1*α* overexpression and nuclear accumulation. Furthermore, NOX4 depleted can rescue the effect of the TGF-*β*1-treatment on PI3K/AKT/HIF-1*α*. Another evidence that supports the function of nuclear accumulation for HIF-1*α* is the change of mRNA levels of downstream molecules. It should be noted that the generation of ROS is known to stabilize HIF-1*α* under various conditions [46, 48]. In agreement with these investigations, we observed that HIF-1*α* mRNA levels have no significant changes whenever treated with TGF-*β*1 or NOX4 and PI3K/AKT suppression. Hence, further investigations are still required to determine the role of ROS in the transcription, accumulation, and stabilization of HIF-1*α*. Altogether, these observations give a more in-depth understanding of the oncogenic molecular mechanism through which the TGF-*β*1/NOX4/ROS axis promotes glioblastoma progression and can provide clues for the antimetabolism treatment of glioblastoma.

Thus, targeting TGF-*β*1 directly and its downstream signaling but not be limited to accompanying metabolic rewiring during EMT might provide a potential window for cancer treatment.

## 5. Conclusions

Collectively, we identified for the first time that TGF-*β*1 stimulated NOX4-dependent ROS expression through the Smad signal pathway, which induced metabolic reprogramming and EMT in glioblastoma and showed an uncharacterized link between tumor microenvironment, metabolism, and EMT in glioblastoma holds promising strategies for cancer therapy.

## Figures and Tables

**Table 1:** Prime sequences.

Genes	Sequence
TGF-*β*1	Forward: 5′-GCAACAATTCCTGGCGATACC-3′ Reverse: 5′-ATTTCCCCTCCACGGCTCAA-3′

NOX4	Forward: 5′-CTGGAGGAGCTGGCTCGCCAACGAAG-3′ Reverse: 5′-GTGATCATGAGGAATAGCACCACCACCATGCAG-3′

GLUT1	Forward: 5′-AACCACTGCAACGGCTTAGA-3′ Reverse: 5′-TCACGGCTGGCACAAAACTA-3′

HK2	Forward: 5′-CCTGTGGCTTTFAAGACCT-3′ Reverse: 5′-CATGTTCACACACATCCGCC-3′

LDHA	Forward: 5′-TTCAGCCCGATTCCGTTACC-3′ Reverse: 5′-CAAGGACCCACCCATGACAG-3′

PDK1	Forward: 5′-GCAAATCACCAGGACAGCC-3′ Reverse: 5′-ACCCAGCGTGACATGAACTT-3′

HIF-1*α*	Forward: 5′-TGCTTTAACTTTGCTGGCCC-3′ Reverse: 5′-GTTTCTGTGTCGTTGCTGCC-3′

N-Cadherin	Forward: 5′-GTGCATGAAGGACAGCCTCT-3′ Reverse: 5′-CCACCTTAAAATCTGCAGGC-3′
*β*-Actin	Forward: 5′-CACCATTGGCAATGAGCGGTTC-3′ Reverse: 5′-AGGTCTTTGCGGATGTCCACGT-3′

**Table 2:** siRNA sequences.

Genes	Sequences
Nontargeting siRNA	siRNA sense: 5′-UUCUCCGAACGUGUCACGUTT-3′ Antisense: 5′-ACGUGACACGUUCGGAGAATT-3′

Smad3	siRNA sense: 5′-GCCUGGUCAAGAAACUCAATT-3′ Antisense: 5′-UUGAGUUUCUUGACCAGGCTT-3′

LDHA	siRNA sense: 5′-CUGGCAAAGACUAUAAUGUTT-3′ Antisense: 5′-ACAUUAUAGUCUUUGCCAGTT-3′

PDK1	siRNA sense: 5′-GCCAAUACAAGUGGUUUAUTT-3′ Antisense: 5′-AUAAACCACUUGUAUUGGCTT-3′

HIF-1*α*	siRNA sense: 5′-CUGAUGACCAGCAACUUGA-3′ Antisense: 5′-UCAAGUUGCUGGUCAUCAG-3′

## Data Availability

The data used to support the findings of this study are available from the corresponding author upon request.

## Supplementary Materials

Supplementary Materials Figure S1: (a) quantitative analysis of western blot in Figure 1(c). Figure S2: (a) quantitative analysis of western blot in Figure 2(c); (b) quantitative analysis of western blot in Figure 2(d); (c) quantitative analysis of western blot in Figure 2(e); (d) levels of NOX4 proteins in glioblastoma cells treated for 24 hours with GKT137831 (10 *μ*M); (e) glioblastoma cells were labeled with MitoTracker Red and stained with NOX4 antibody followed by FITC-linked rabbit anti-rabbit secondary antibody. Nuclei were counter stained with DAPI. Scale bar = 50 *μ*m. Figure S3: (a) quantitative analysis of western blot in Figure 3(a); (b) glioblastoma cells transfected with control shRNA or NOX4-specific shRNA lentivirus and western blot analysis for the levels of NOX4 protein expression; (c) quantitative analysis of western blot in Figure 3(c); (d) western blot analysis showed the metabolic enzymes treated with TGF-*β*1 with or without NAC (5 mM) on glioblastoma cells; (e) quantitative analysis of western blot in Figure 3(k). Figure S4: (a) quantitative analysis of western blot in Figure 4(a); (b) quantitative analysis of western blot in Figure 4(b). Figure S5: (a) quantitative analysis of western blot in Figure 5(c); (b) quantitative analysis of western blot in Figure 5(h). Figure S6: (a) quantitative analysis of western blot in Figure 6(a); (b) quantitative analysis of western blot in Figure 6(c); (c) quantitative analysis of western blot in Figure 6(d); (d) the expression of indicated proteins was detected by western blot when HIF-1*α* was deleted under TGF-*β*1 treatment for 24 hours; (e) qPCR analysis of the mRNA levels of HIF-1*α* in the glioblastoma cells treated with TGF-*β*1 or MK-2206 for 24 hours; (f) western blot analysis showed the impact of AKT inhibitor MK-2206 and the HIF-1*α* inhibitor PX-478 on NOX4 protein expression; (g) U87 cells were treated with TGF-*β*1 and TGF-*β*1 plus MK-2206 or PX-478 for 24 hours before being adhered to microplates, and extracellular acidification rate (ECAR) and oxygen consumption rate (OCR) were determined over time and analyzed as bar graphs. Figure S7: (a) quantitative analysis of western blot in Figure 7(e) (Supplementary Materials).
