# Supplementary material for: NOX4-Derived ROS Mediates TGF-β1-Induced Metabolic Reprogramming during Epithelial-Mesenchymal Transition through the PI3K/AKT/HIF-1α Pathway in Glioblastoma
Source: Oxid Med Cell Longev. 2021 Jun 27;2021:5549047. doi: 10.1155/2021/5549047 (PMC8257383; doi:10.1155/2021/5549047)
Supplement: Supplementary Materials — Figure S1: (a) quantitative analysis of western blot in Figure 1(c). Figure S2: (a) quantitative analysis of western blot in Figure 2(c); (b) quantitative analysis of western blot in Figure 2(d); (c) quantitative analysis of western blot in Figure 2(e); (d) levels of NOX4 proteins in glioblastoma cells treated for 24 hours with GKT137831 (10 μM); (e) glioblastoma cells were labeled with MitoTracker Red and stained with NOX4 antibody followed by FITC-linked rabbit anti-rabbit secondary antibody. Nuclei were counter stained with DAPI. Scale bar = 50 μm. Figure S3: (a) quantitative analysis of western blot in Figure 3(a); (b) glioblastoma cells transfected with control shRNA or NOX4-specific shRNA lentivirus and western blot analysis for the levels of NOX4 protein expression; (c) quantitative analysis of western blot in Figure 3(c); (d) western blot analysis showed the metabolic enzymes treated with TGF-β1 with or without NAC (5 mM) on glioblastoma cells; (e) quantitative analysis of western blot in Figure 3(k). Figure S4: (a) quantitative analysis of western blot in Figure 4(a); (b) quantitative analysis of western blot in Figure 4(b). Figure S5: (a) quantitative analysis of western blot in Figure 5(c); (b) quantitative analysis of western blot in Figure 5(h). Figure S6: (a) quantitative analysis of western blot in Figure 6(a); (b) quantitative analysis of western blot in Figure 6(c); (c) quantitative analysis of western blot in Figure 6(d); (d) the expression of indicated proteins was detected by western blot when HIF-1α was deleted under TGF-β1 treatment for 24 hours; (e) qPCR analysis of the mRNA levels of HIF-1α in the glioblastoma cells treated with TGF-β1 or MK-2206 for 24 hours; (f) western blot analysis showed the impact of AKT inhibitor MK-2206 and the HIF-1α inhibitor PX-478 on NOX4 protein expression; (g) U87 cells were treated with TGF-β1 and TGF-β1 plus MK-2206 or PX-478 for 24 hours before being adhered to microplates, and extracellular acidification rat [file 5549047.f1.docx]

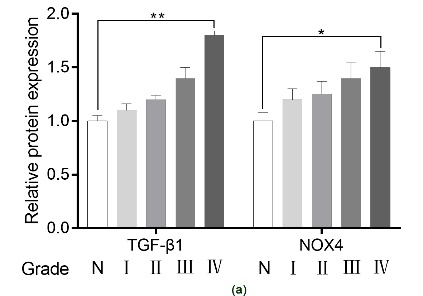


Figure S1: (a) Quantitative analysis of western blot in figure 1(c).


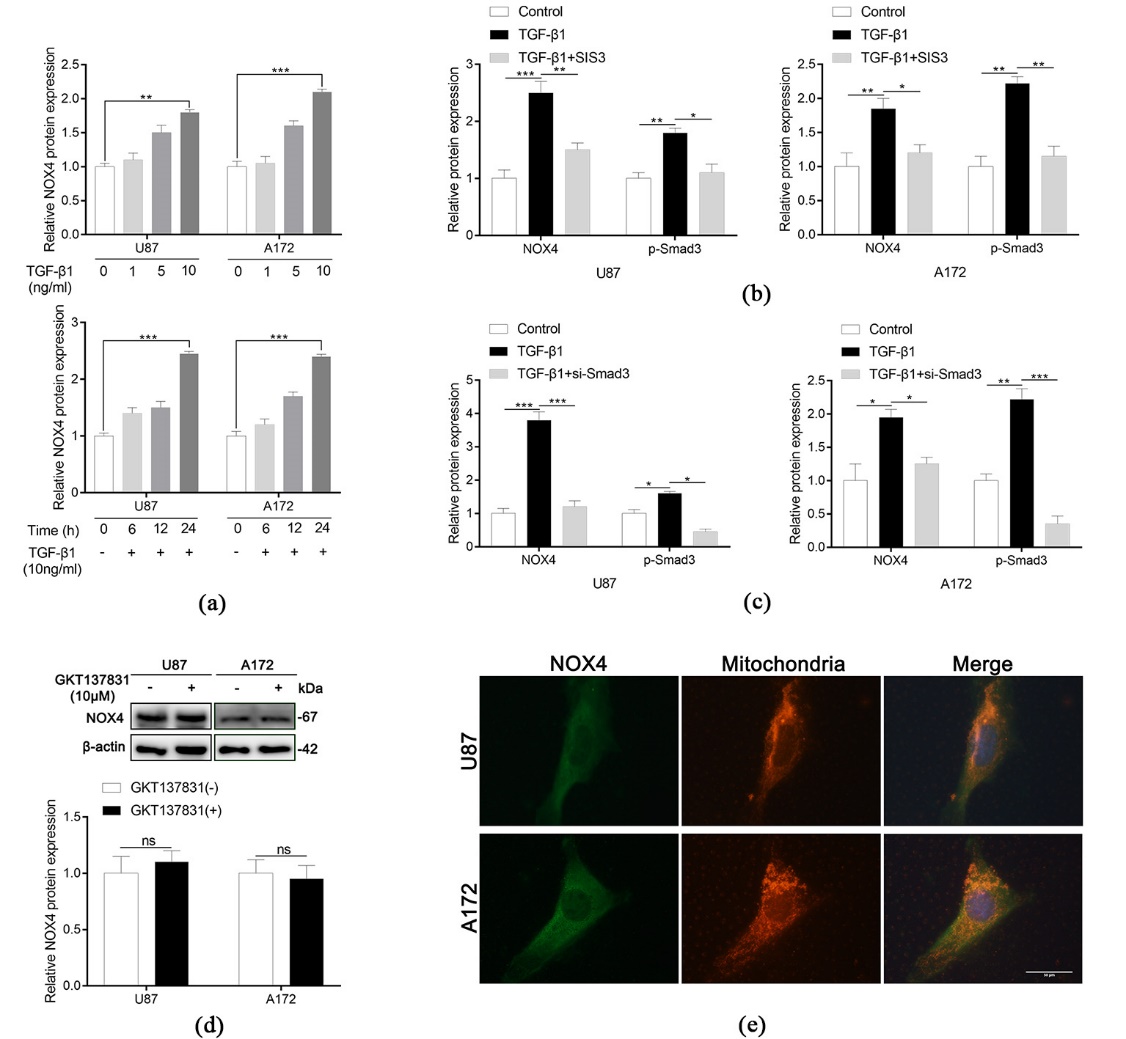


Figure S2: (a) Quantitative analysis of western blot in figure 2(c). (b) Quantitative analysis of western blot in figure 2(d). (c) Quantitative analysis of western blot in figure 2(e). (d) Levels of NOX4 proteins in glioblastoma cells treated for 24 hours with GKT137831 (10 μM). (e) Glioblastoma cells were labeled with MitoTracker red and stained with NOX4 antibody followed by FITC-linked rabbit anti-rabbit secondary antibody. Nuclei were counter stained with DAPI. Scale bar = 50 μm.


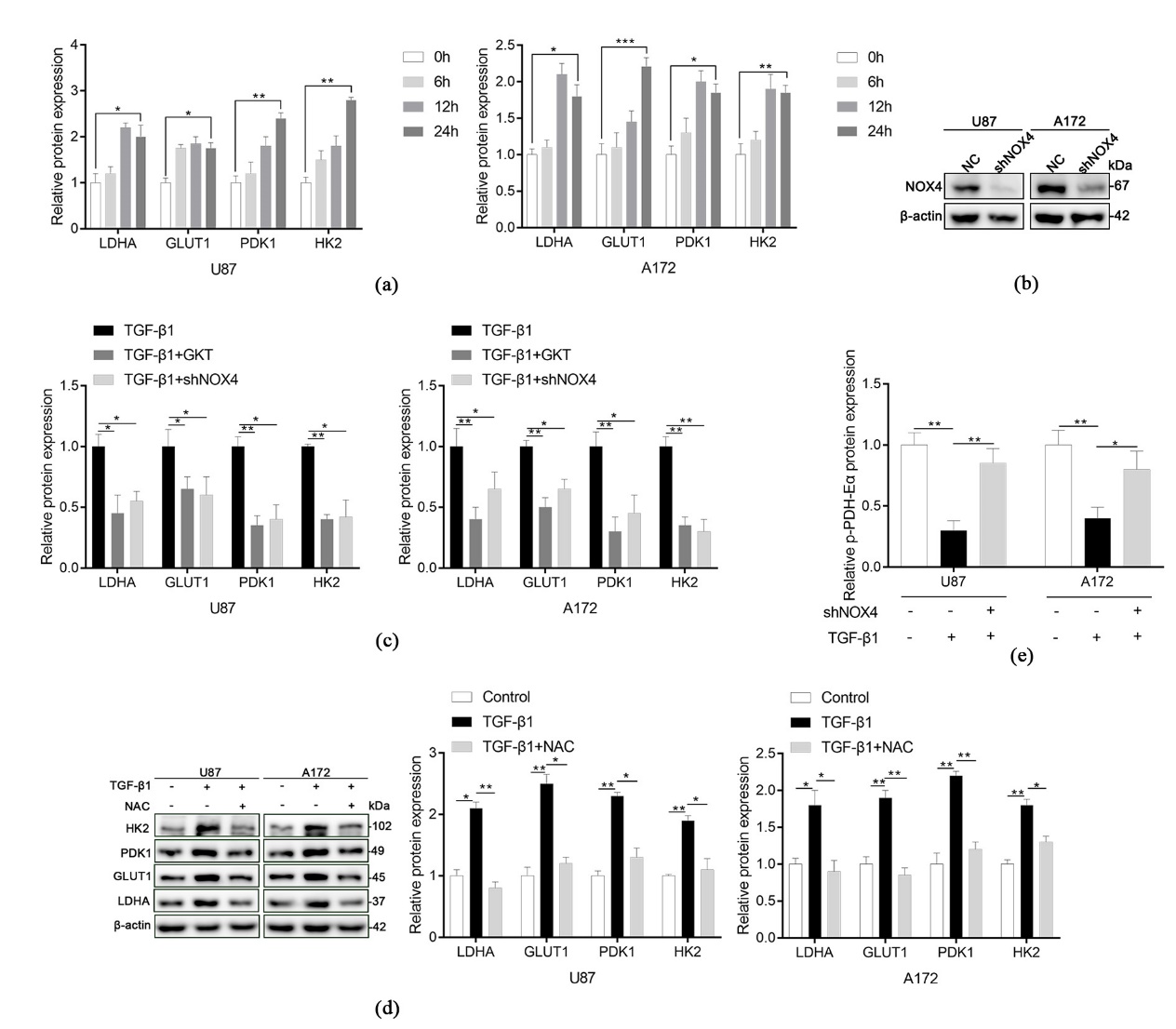


Figure S3: (a) Quantitative analysis of western blot in figure 3(a). (b) Glioblastoma cells transfected with control shRNA or NOX4-specific shRNA lentivirus and western blot analysis for the levels of NOX4 protein expression. (c) Quantitative analysis of western blot in figure 3(c). (d) Western blot analysis showed the metabolic enzymes treated with TGF-β1 with or without NAC (5 mM) on glioblastoma cells. (e) Quantitative analysis of western blot in figure 3(k).


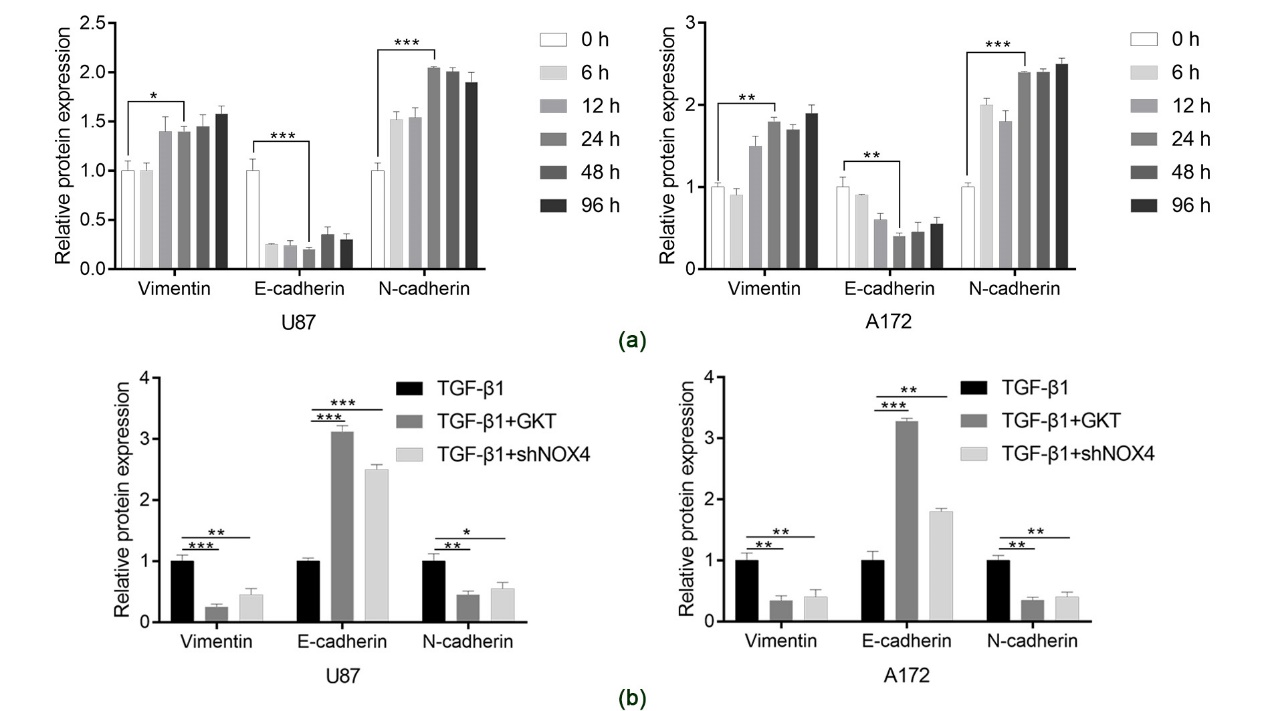
­

Figure S4: (a) Quantitative analysis of western blot in figure 4(a). (b) Quantitative analysis of western blot in figure 4(b).


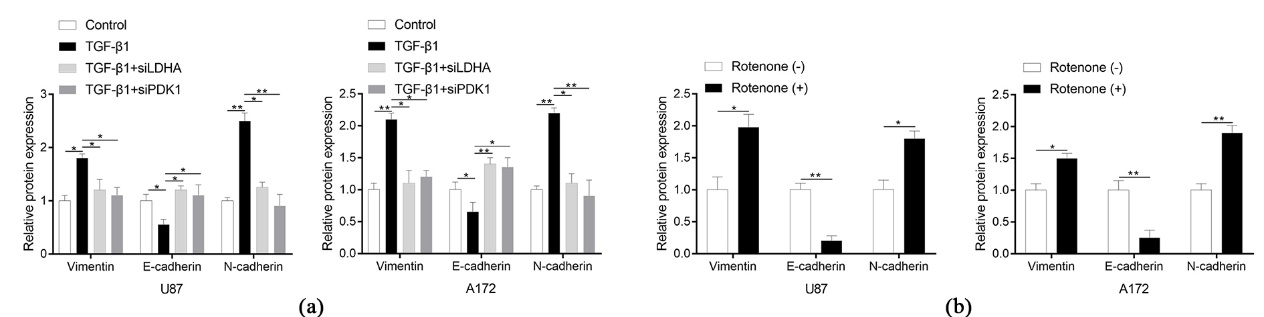


Figure S5: (a) Quantitative analysis of western blot in figure 5(c). (b) Quantitative analysis of western blot in figure 5(h).


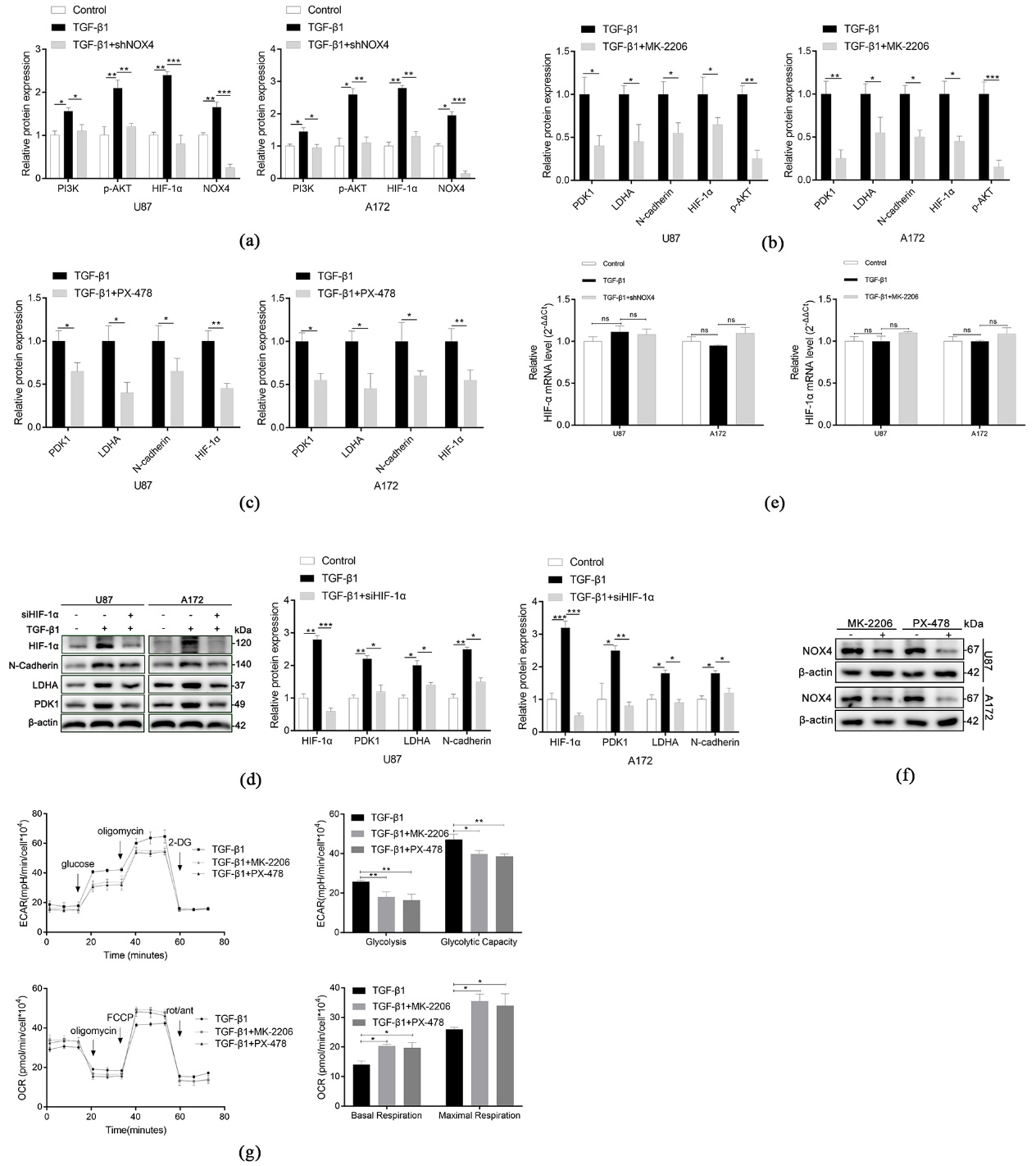


Figure S6: (a) Quantitative analysis of western blot in figure 6(a). (b) Quantitative analysis of western blot in figure 6(c). (c) Quantitative analysis of western blot in figure 6(d). (d) The expression of indicated proteins was detected by western blot when HIF-1α was deleted under TGF-β1 treatment for 24 hours. (e) qPCR analysis of the mRNA levels of HIF-1α in the glioblastoma cells treated with TGF-β1 or MK-2206 for 24 hours. (f) Western blot analysis showed the impact of AKT inhibitor MK-2206 and the HIF-1α inhibitor PX-478 on NOX4 protein expression. (g) U87 cells were treated with TGF-β1 and TGF-β1 plus MK-2206 or PX-478 for 24 hours before being adhered to microplates, and extracellular acidification rate (ECAR), and oxygen consumption rate (OCR) were determined over time and analyzed as bar graphs.


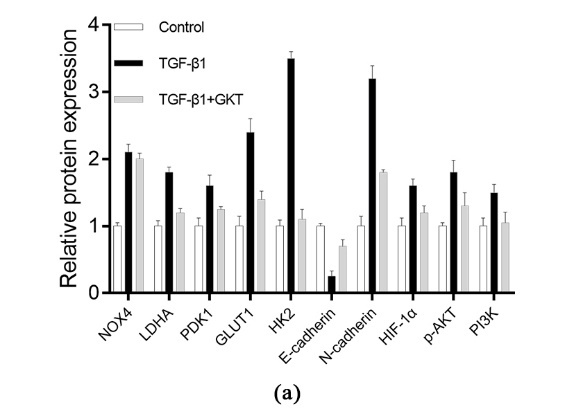


Figure S7: (a) Quantitative analysis of western blot in figure 7(e).
